# Initiation and Development of Nurse Practitioner Practice in a Japanese Rehabilitation Hospital: A Mixed-Methods Descriptive Study

**DOI:** 10.7759/cureus.103330

**Published:** 2026-02-10

**Authors:** Ryoko Yamauchi, Ryuichi Ohta, Kengo Kato, Chiaki Sano

**Affiliations:** 1 Community Medicine Management, Shimane University Faculty of Medicine, Izumo, JPN; 2 Community Care, Unnan City Hospital, Unnan, JPN; 3 Critical Care Medicine, Tokyo Metropolitan Bokutoh Hospital, Tokyo, JPN

**Keywords:** delivery of health care, interprofessional relations, japan, nurse practitioners, post-acute care, rehabilitation hospitals

## Abstract

Introduction

Nurse practitioners (NPs) in Japan are primarily concentrated in acute care settings, with limited integration into rehabilitation hospitals. As Japan’s healthcare system increasingly emphasizes chronic and community-based care, understanding NP roles in post-acute rehabilitation is essential. This study aimed to examine the initiation and development of NP practice in a Japanese rehabilitation hospital, focusing on the scope, processes, and patterns of clinical activities.

Methods

We conducted a mixed-methods descriptive study at Kanagawa Rehabilitation Hospital over 12 months (April 2023-March 2024). All NP-involved clinical activities were extracted from hospital records, including the date, care setting, department, initiation mode, and free-text intervention descriptions. Quantitative data were analyzed using descriptive statistics, while qualitative content analysis identified thematic domains of NP practice.

Results

A total of 738 NP activities were recorded, most occurring in adult inpatient wards (n = 623, 84.4%), followed by pediatrics (n = 87, 11.8%) and clerical/administrative contexts (n = 28, 3.8%). Activities were concentrated in internal medicine (30.5%) and orthopedic (29.7%) departments. Over half of all interventions were self-initiated (415, 56.2%), indicating substantial professional autonomy. Qualitative analysis revealed four primary domains: direct clinical management (e.g., medication adjustments, swallowing care), care coordination (e.g., multidisciplinary conferences, interdepartmental referrals), patient and family education (e.g., disease-specific counseling, self-management guidance), and administrative/quality improvement activities (e.g., order entry, safety initiatives).

Conclusion

This study indicates that NP practice in rehabilitation settings encompasses diverse clinical, coordination, educational, and administrative roles, reflecting a high level of professional autonomy and integration into multidisciplinary care. These results provide insight into the scope and characteristics of NP practice in post-acute rehabilitation settings in Japan. Future studies are needed to evaluate the impact of NP integration on measurable patient and system-level outcomes, including quality, efficiency, and continuity of care.

## Introduction

In Japan, the education and professional integration of nurse practitioners (NPs) are still in their developmental stages [1,2]. While the number of certified NPs is gradually increasing, their roles remain diverse and are most concentrated in acute care settings [3-5]. In Japan, NPs are registered nurses who complete a graduate-level NP education program accredited by the Japanese Organization of Nurse Practitioner Faculties, which typically requires completion of a master’s degree and includes advanced coursework in clinical assessment, pharmacology, and pathophysiology, as well as supervised clinical training [6,7]. Upon completion of the program and certification process, individuals are recognized as certified NPs. However, NP practice in Japan is not yet fully regulated under national legislation, and their scope of practice is defined primarily by institutional policies and physician collaboration [6,8]. However, as Japan’s healthcare system faces rising demands for chronic and community-based care, there is growing interest in expanding NP practice into subacute and long-term care contexts, including rehabilitation and primary care [3].

Rehabilitation hospitals in Japan primarily serve patients with high medical dependency, often following hospitalization for acute events such as stroke, trauma, or surgery [4]. These facilities, which offer extended inpatient rehabilitation, represent a crucial component of post-acute care. Despite this, rehabilitation hospitals in Japan typically operate under a physician-led model in which rehabilitation physicians and ward-based physicians are primarily responsible for medical management, while multidisciplinary teams, including nurses, physical therapists, occupational therapists, and speech therapists, provide rehabilitative interventions. However, the integration of advanced practice providers such as NPs remains limited, and medical decision-making responsibilities are largely concentrated among physicians [5].

Given their training in comprehensive clinical assessment, care coordination, and patient education, NPs may be uniquely positioned to contribute to the improvement of care quality in rehabilitation hospitals [6,7]. Their involvement could also enhance patient outcomes and staff collaboration, particularly in managing medically complex patients requiring continued rehabilitation [8].

To date, however, there is a paucity of research examining the practice of NPs in rehabilitation hospitals in Japan. Understanding their roles, clinical activities, and working processes in these settings could provide valuable insights into optimizing their contributions, improving care integration, and informing policy on the deployment of the NP workforce. This study aims to explore the initiation and development of NP practice in Japanese rehabilitation hospitals and to elucidate the specific clinical roles and processes involved in their daily work through a mixed-methods approach.

## Materials and methods

We conducted a mixed-methods descriptive study to explore the initiation and development of NP's practice in rehabilitation hospitals in Japan. This study was carried out in a single rehabilitation hospital that primarily provides post-acute inpatient care for adults with high medical dependency, including those recovering from stroke, trauma, or surgery.

Setting and participants

The study was conducted at the Kanagawa Rehabilitation Hospital, located in Atsugi City, Kanagawa Prefecture, Japan. Established in 1973, this center serves as a prefectural core rehabilitation hospital with approximately 320 beds. It offers comprehensive rehabilitation services and collaborates with social welfare facilities and regional rehabilitation support centers to provide integrated and continuous care. The hospital encompasses numerous departments, including orthopedic surgery, neurosurgery, internal medicine, surgery, urology, neurology, pediatrics, ophthalmology, ENT, dermatology, dental/oral surgery, anesthesiology, and specialized rehabilitation disciplines such as physical therapy, occupational therapy, speech therapy, psychology, sports therapy, vocational therapy, and rehabilitation engineering.

Before this study, there was minimal integration of NPs into the clinical workflow. In April 2022, one certified NP began practice at the hospital. The NP had over 15 years of clinical experience as a registered nurse, including 10 years in acute care settings such as internal medicine and perioperative care. The NP had completed a master’s-level NP education program accredited by the Japanese Organization of Nurse Practitioner Faculties and had obtained national certification prior to joining the hospital. Before initiating independent clinical activities, the NP underwent institutional orientation specific to the rehabilitation setting, including familiarization with rehabilitation workflows, multidisciplinary team structures, and common medical issues in post-acute care patients. All clinical activities involving this NP during the first year were included in the analysis.

Although the NP’s prior clinical experience was primarily in adult acute care settings, pediatric-related activities were conducted within the structured rehabilitation care system and did not involve independent pediatric specialty management. The NP received institutional orientation, including familiarization with pediatric rehabilitation workflows and multidisciplinary collaboration processes. Pediatric care activities were performed in collaboration with attending physicians and rehabilitation specialists, with appropriate supervision.

Data collection

We retrospectively extracted NP activity data from hospital records covering the period from April 3, 2023, to March 31, 2024. The dataset was derived from routine clinical activity documentation recorded by the NP in the hospital’s electronic medical record system as part of standard clinical practice. The NP documented each clinical activity contemporaneously in accordance with institutional documentation protocols. For this study, activity data were retrospectively extracted from the electronic medical record using predefined variables, including timing (date of activity), anonymized patient identifier, care setting (e.g., adult inpatient ward), department (e.g., neurology, internal medicine), job orientation (e.g., self-initiated, routine, request-based), and intervention description (e.g., comprehensive clinical assessment, medication adjustment, care coordination, patient and family education, and participation in multidisciplinary conferences).

Quantitative analysis

Descriptive statistical analyses were performed using EZR (Saitama Medical Center, Jichi Medical University, Saitama, Japan), a graphical user interface for R. Frequencies and percentages were calculated to characterize NP activities by clinical department, type of intervention, care setting, and mode of initiation (self-initiated, routine, or request-based).

Qualitative analysis

For the qualitative component, we conducted a thematic content analysis of all free-text intervention descriptions documented in the NP activity records [9]. The analysis followed an inductive approach, allowing themes to emerge directly from the data rather than applying a pre-existing framework. Initially, two researchers (RY and RO) independently read the entire dataset multiple times to become familiar with its content and context. RY is a certified NP with clinical experience in rehabilitation and acute care settings and formal training in NP practice and research. RO is a physician specializing in general medicine and community-based care, with extensive experience in NP research, qualitative research methodologies, and interdisciplinary healthcare systems. Both researchers have prior experience conducting qualitative studies using thematic analysis. They then conducted open coding, identifying and labeling discrete units of meaning within each intervention record. Codes reflected observed actions, processes, and contextual elements of NP practice.

After the initial coding, the two researchers compared their code lists to identify overlaps and differences. Discrepancies in code interpretation or application were resolved through in-depth discussion between the two researchers, with repeated reference to the original intervention text to ensure data fidelity. To enhance the credibility and dependability of the analysis, both researchers independently conducted initial coding and subsequently compared their coding frameworks. Consensus was reached through iterative discussion, and all coding decisions were documented throughout the analytic process to ensure transparency and consistency. This process enhanced inter-coder reliability. Once consensus was reached on the coding framework, all intervention records were re-examined and recoded as necessary to maintain consistency.

The finalized codes were then organized into categories representing recurrent patterns of NP activities. These categories were subsequently mapped to broader role domains: direct clinical care, care coordination, patient and family education, and administrative and quality improvement activities. Each domain was defined and supported by representative examples from the data to illustrate the scope and variability of NP contributions in the rehabilitation hospital context.

Ethics

The study protocol was approved by the Clinical Ethics Committee of Unnan City Hospital (approval number: 20250003), which served as the primary institutional review board for this study because the principal investigator was affiliated with Unnan City Hospital. The study was conducted in collaboration with Kanagawa Rehabilitation Hospital, where the clinical activity data were collected. All patient identifiers were anonymized prior to analysis to ensure confidentiality.

## Results

During the 12-month observation period from April 2023 to March 2024, a total of 738 NP-involved clinical activities were documented at the Kanagawa Rehabilitation Hospital. Most activities took place in the adult inpatient rehabilitation wards (n = 623, 84.4%), which typically accommodate patients recovering from acute conditions such as stroke, major orthopedic injuries, and postoperative complications. This high proportion reflects the hospital’s core mission of providing extended, multidisciplinary post-acute care to medically complex adult patients.

Pediatric wards accounted for 87 activities (11.8%), involving children with long-term rehabilitation needs following neurological, orthopedic, or congenital conditions. In these cases, the NP’s role often included developmental assessments, family education, and coordination with community-based pediatric services.

Clerical and administrative contexts comprised 28 activities (3.8%), reflecting the NP’s involvement in institutional operations such as documentation, safety checks, and participation in hospital management initiatives. These activities demonstrate the integration of NP practice not only into bedside care but also into organizational processes that support quality improvement and care continuity. Care setting and clinical department represent two distinct classification dimensions. Care setting refers to the physical or organizational context in which the activity occurred (e.g., adult inpatient ward, pediatric ward), whereas clinical department refers to the primary medical specialty responsible for the patient’s care (e.g., internal medicine, orthopedic surgery). Each NP activity was categorized once within each dimension, and no double-counting occurred (Table 1).

**Table 1:** Distribution of Nurse Practitioner Activities by Care Setting (April 2023–March 2024) Percentages are calculated from the total number of NP-involved activities (n = 738) recorded between April 2023 and March 2024. “Adult inpatient wards” refer to rehabilitation units for adult patients recovering from acute conditions such as stroke, orthopedic trauma, or surgery. “Pediatric wards” include long-term rehabilitation for children with neurological, orthopedic, or congenital conditions. “Clerical/administrative” includes activities outside direct patient care, such as documentation, safety management, and participation in institutional operations.

Care Setting	n	%	Description of NP Role
Adult inpatient wards	623	84.4%	Care for medically complex adults’ post-stroke, orthopedic injury, and postoperative recovery.
Pediatric wards	87	11.8%	Developmental assessment, family education, and coordination with community pediatric services.
Clerical/administrative	28	3.8%	Documentation, safety checks, and participation in hospital management initiatives.

Distribution by department

NP activities were most frequently recorded in internal medicine (n = 225, 30.5%) and orthopedic departments (n = 219, 29.7%), together accounting for more than 60% of all recorded interventions. In the internal medicine wards, activities often involve managing patients with comorbidities such as diabetes mellitus, chronic heart failure, and post-stroke complications, requiring frequent medication adjustments, glycemic control, and monitoring for secondary prevention. In orthopedic wards, the NP’s role frequently focused on perioperative management, prevention of postoperative complications, wound care, and coordination of rehabilitation plans to facilitate functional recovery.

Other notable activity areas included pediatrics (n = 95, 12.9%), where the NP engaged in care for children requiring long-term rehabilitation following neurological injury, congenital disorders, or orthopedic surgery. Anesthesiology-related activities (n = 92, 12.5%) primarily involved perioperative medical management within the rehabilitation setting. These included preoperative clinical assessments, postoperative monitoring, pain management support, and coordination with anesthesiologists for patients undergoing procedures such as pressure ulcer surgery and orthopedic interventions. These activities were conducted in collaboration with the anesthesiology and surgical teams. The difference between pediatric ward activities (n = 87) and pediatric department activities (n = 95) reflects the distinction between care setting and clinical department classification. While pediatric wards refer to the physical location of care delivery, the pediatric department classification refers to the clinical service responsible for the patient’s management. Some pediatric department activities occurred in shared rehabilitation settings rather than exclusively in pediatric wards.

In rehabilitation medicine (n = 32, 4.3%), NP activities included interdisciplinary goal-setting conferences, functional status assessments, and monitoring of medical stability during therapy. Management-related activities (n = 24, 3.3%) reflected involvement in hospital-wide initiatives such as safety checks, quality improvement meetings, and policy discussions.

Smaller but clinically relevant contributions were noted in nursing administration (n = 20, 2.7%) through staff education and workflow optimization, urology (n = 13, 1.8%) for management of catheter-related issues and postoperative follow-up, and neurology (n = 12, 1.6%) for evaluation and management of complex neurorehabilitation cases. Isolated but specialized activities were also recorded in neurosurgery (n = 3, 0.4%), surgical operations (n = 2, 0.3%), and clerical services (n = 1, 0.1%) (Table 2).

**Table 2:** Distribution of Nurse Practitioner Activities by Department (April 2023–March 2024) Percentages are calculated from the total NP-involved activities (n = 738). Department classification reflects the primary clinical service responsible for the patient’s care and represents a separate classification dimension from care setting. Each activity was assigned to a single department, and no double-counting occurred.

Department	n	%	Description of NP Role
Internal medicine	225	30.5%	Management of comorbidities (e.g., diabetes, chronic heart failure, post-stroke care), medication adjustments, glycemic control, and secondary prevention monitoring.
Orthopedic	219	29.7%	Perioperative management, prevention of postoperative complications, wound care, and coordination of rehabilitation plans.
Pediatrics	95	12.9%	Long-term rehabilitation for neurological injury, congenital disorders, or orthopedic surgery; developmental assessment; family education.
Anesthesiology	92	12.5%	Perioperative assessments, anesthesia risk evaluation, postoperative pain management.
Rehabilitation medicine	32	4.3%	Interdisciplinary goal-setting conferences, functional status assessment, and medical stability monitoring during therapy.
Management	24	3.3%	Safety checks, quality improvement meetings, policy development, and hospital-wide initiatives.
Nursing administration	20	2.7%	Staff education, workflow optimization, and nursing operations support.
Urology	13	1.8%	Catheter-related care and postoperative follow-up.
Neurology	12	1.6%	Complex neurorehabilitation assessments and management.
Neurosurgery	3	0.4%	Specialized perioperative and postoperative neurosurgical care.
Surgical operations	2	0.3%	Intraoperative or perioperative support.
Clerical services	1	0.1%	Administrative support unrelated to direct clinical care.

Job orientation and initiation mode

Most NP activities were self-initiated (n = 415, 56.2%), indicating a high degree of professional autonomy and the ability to proactively identify and address clinical needs without direct instruction. This reflects the NP’s integration into daily clinical operations as a trusted advanced practice provider. Routine scheduled work accounted for 196 activities (26.6%), ensuring continuity of care for patients with predictable rehabilitation and medical needs and providing opportunities for the NP to standardize care processes across departments.

Activities initiated by nurses (n = 67, 9.1%) were often related to acute changes in patient condition, complex wound care, or clarification of treatment plans, highlighting the NP’s role as an accessible clinical resource for nursing staff. Requests from physicians (n = 48, 6.5%) typically involved targeted assessments, procedural preparation, or assistance in managing complex medical cases.

Although less frequent, community association requests (n = 5, 0.7%) reflected the NP’s emerging role in bridging inpatient care with community-based services, particularly in discharge planning and continuity of rehabilitation after hospitalization. Very infrequent initiation by nutritionists (n = 2, 0.3%), safety officers (n = 2, 0.3%), and patients themselves (n = 2, 0.3%) demonstrated the NP’s flexibility to contribute to diverse aspects of patient care and institutional safety. Only one activity (0.1%) was initiated by an anesthesiologist, suggesting occasional cross-specialty collaboration in specific perioperative scenarios (Table 3).

**Table 3:** Nurse Practitioner Activities by Job Orientation and Initiation Mode (April 2023–March 2024) Percentages are calculated from the total NP-involved activities (n = 738). “Self-initiated” refers to activities undertaken by the NP without external prompting. Other categories denote the primary source initiating the NP activity.

Initiation Mode	n	%	Description of NP Role
Self-initiated	415	56.2%	Independent patient assessments, medication adjustments, and multidisciplinary care coordination without direct instruction.
Routine scheduled work	196	26.6%	Regular ward rounds, participation in pre-scheduled conferences, follow-up evaluations, and standardized care processes.
Nurse request	67	9.1%	Response to acute patient condition changes, complex wound care needs, and treatment plan clarification.
Physician request	48	6.5%	Targeted assessments, procedural preparation, and assistance in complex case management.
Community association	5	0.7%	Coordination with community services for discharge planning and post-hospital rehabilitation continuity.
Nutritionist request	2	0.3%	Nutritional care input and collaboration in patient management.
Safety officer request	2	0.3%	Institutional safety checks and preventive measures.
Patient request	2	0.3%	Direct patient-initiated consultations and care requests.
Anesthesiologist request	1	0.1%	Cross-specialty collaboration in perioperative patient care.

Qualitative analysis of intervention types

Content analysis of the 738 free-text intervention records identified four primary domains of NP practice: direct clinical management, care coordination, patient and family education, and administrative and quality improvement activities. Each activity was assigned to a single primary domain based on its principal clinical purpose. Direct clinical management represented the largest domain, followed by care coordination, patient and family education, and administrative and quality improvement activities. These findings highlight the broad and integrative scope of NP practice in the rehabilitation setting.

Direct Clinical Management

This domain encompassed bedside clinical assessments, medication adjustments, and preparation for diagnostic or therapeutic procedures. Examples included swallowing management and aspiration prevention for a post-stroke patient; insulin titration for glycemic control during neurorehabilitation; postoperative wound care combined with metabolic control; perioperative medication adjustments and patient preparation prior to pressure ulcer surgery; and integration of swallowing evaluation with tube feeding management for a patient with dysphagia.

Care Coordination

NPs frequently facilitated communication and alignment across departments and with community-based care providers. Examples included leading case presentations and treatment discussions during internal medicine conferences; coordinating multidisciplinary interventions for medically unstable patients with poor appetite, nausea, and vomiting after surgery; and aligning perioperative planning between surgical and medical teams to ensure smooth transitions in care.

Patient and Family Education

Educational activities were often embedded within direct care. Examples included providing instructions on medication changes and infusion therapy after pressure ulcer surgery; counseling patients and families on intensive insulin therapy and self-monitoring techniques; and reinforcing blood glucose monitoring protocols for patients undergoing post-stroke rehabilitation.

Administrative and Quality Improvement Activities

NPs also contributed to the operational and safety infrastructure of the hospital. Examples included entering and verifying physician orders for complex postoperative cases, discontinuing infusion therapy with appropriate documentation, and supporting medication adjustment processes by integrating clinical findings with administrative workflows.

Summary of qualitative analysis

These examples demonstrate that NP practice in the rehabilitation hospital was multifaceted, integrating direct patient care with coordination, education, and system-level activities. The frequent overlap between domains, such as combining clinical management with patient education and administrative support, highlights the adaptive and integrative nature of NP roles in this context (Table 4).

**Table 4:** Qualitative Analysis of Nurse Practitioner Activities by Domain (April 2023–March 2024) Domains were derived from thematic content analysis of 738 NP activity records. Examples are anonymized and represent typical activities within each domain.

Domain	Description	Representative Examples
Direct Clinical Management	Bedside assessments, medication adjustments, and preparation for diagnostic/therapeutic procedures.	Swallowing management and aspiration prevention for a post-stroke patient; insulin titration during neurorehabilitation; postoperative wound care with metabolic control; perioperative drug adjustments and preparation for pressure ulcer surgery; integration of swallowing evaluation with tube feeding management.
Care Coordination	Facilitating communication and alignment across departments and with community care providers.	Leading case presentations and treatment discussions in internal medicine conferences; coordinating multidisciplinary interventions for medically unstable postoperative patients; aligning perioperative planning between surgical and medical teams.
Patient and Family Education	Delivering disease-specific education and self-management guidance, often within direct care.	Instructions on medication changes and infusion therapy after pressure ulcer surgery; counseling on intensive insulin therapy and self-monitoring; reinforcing blood glucose monitoring protocols for post-stroke patients.
Administrative and Quality Improvement Activities	Supporting hospital operations, safety, and workflow optimization.	Entering and verifying physician orders for complex postoperative cases; discontinuing infusion therapy with proper documentation; integrating clinical findings into administrative processes for medication adjustments.

## Discussion

This study provides one of the first detailed examinations of NP practice in a Japanese rehabilitation hospital, demonstrating both the breadth and depth of their contributions across clinical, educational, coordination, and administrative domains. The findings highlight that NPs can play a central role in managing medically complex patients in post-acute settings, bridging gaps between acute and community care, and enhancing multidisciplinary collaboration.

The predominance of self-initiated activities in this study indicates that the NP demonstrated the ability to identify and address patient needs proactively, exercising a level of clinical autonomy that remains relatively uncommon in Japanese healthcare settings, where NP roles are still evolving and lack a standardized definition. This finding suggests that, when integrated into the care team, NPs can contribute meaningfully beyond delegated tasks, initiating interventions based on clinical judgment and patient assessment [10]. Such autonomy is consistent with evidence from international studies, which have shown that NPs can enhance patient outcomes, improve care efficiency, and support multidisciplinary teams through independent clinical decision-making and comprehensive case management, particularly in environments managing medically complex patient populations [11,12].

The distribution of NP activities, with a strong concentration in internal medicine and orthopedics, mirrors the typical patient profile of rehabilitation hospitals, where care frequently focuses on recovery from stroke and orthopedic trauma, as well as management of complex comorbidities. This concentration highlights the important role of NPs in supporting the medical management of medically complex rehabilitation patients and facilitating multidisciplinary care coordination [13,14]. By assisting with clinical assessment, treatment adjustments, and care coordination, NPs may help reduce physician workload and enhance the efficiency and continuity of care delivery in rehabilitation settings. Notably, the substantial proportion of activities involving anesthesiology and pediatrics further demonstrates the adaptability of NP practice across diverse clinical specialties [15]. In these contexts, NP involvement may help address increasing clinical demands, support perioperative management, and enhance team-based care, particularly in settings with limited physician availability [16].

Qualitative findings further demonstrate that the NP’s contributions extended beyond bedside clinical care to include care coordination, patient and family education, and participation in hospital-wide quality improvement initiatives. This integrated scope of practice supports multidisciplinary collaboration and enhances care delivery across clinical settings [17]. Previous studies have shown that collaboration between NPs and physicians can improve care continuity, enhance patient satisfaction, and optimize healthcare efficiency by supporting timely clinical interventions and reducing physician workload. Such collaborative practice models are particularly valuable in rehabilitation hospitals, where multidisciplinary coordination is essential for managing medically complex patients [18].

The importance of NP integration is further underscored by increasing healthcare demands associated with population aging and the rising prevalence of chronic and disabling conditions requiring rehabilitation. In Japan, the proportion of older adults continues to increase, contributing to greater demand for post-acute and rehabilitation services. These demographic and workforce trends highlight the potential value of NPs in supporting healthcare teams, enhancing care coordination, and addressing workforce limitations in rehabilitation settings. By bridging disciplinary gaps and supporting ongoing patient management, NPs may contribute to more efficient, sustainable, and patient-centered rehabilitation care delivery.

Our results also have important implications for the NP workforce policy in Japan. Japan is experiencing rapid population aging, with individuals aged 65 years and older comprising nearly one-third of the population, leading to increasing demand for rehabilitation and chronic disease management. At the same time, healthcare systems face workforce challenges, including physician shortages and increasing clinical workload associated with the growing prevalence of complex, chronic conditions. These trends highlight the need for expanded multidisciplinary workforce models to ensure sustainable healthcare delivery.

The number of NPs in Japan has steadily increased over the past decade as graduate-level NP education programs have expanded. However, their integration into rehabilitation and subacute care settings remains limited [19,20]. Several barriers may contribute to this gap, including the absence of national regulatory frameworks defining the NP scope of practice, variability in institutional implementation, and limited awareness of NP roles among healthcare organizations.

To support effective NP integration, institutional and policy-level efforts are needed, including the development of standardized education and training pathways, the establishment of clear clinical roles and scope of practice, and the implementation of supportive clinical infrastructure that enables interdisciplinary collaboration. With appropriate institutional and regulatory support, NPs have the potential to enhance healthcare workforce capacity, improve care coordination, and contribute to sustainable healthcare delivery in aging societies.

Several limitations should be considered when interpreting these findings. First, this was a single-site study involving one NP, which may limit the generalizability of the results to other rehabilitation hospitals with different organizational cultures or patient populations. Second, the study relied on routinely collected activity logs, which may not fully capture informal or unrecorded NP contributions, particularly in areas such as informal staff mentoring or ad hoc consultations. Third, the qualitative analysis was based on intervention descriptions that varied in detail, potentially influencing the depth of thematic coding. Finally, the observational design precludes direct measurement of the NP’s impact on patient outcomes, such as functional recovery, readmission rates, or patient satisfaction.

## Conclusions

This mixed-methods study demonstrates that an NP working in a Japanese rehabilitation hospital can make substantial contributions across multiple domains, including direct clinical management, care coordination, patient and family education, and administrative and quality improvement activities. The predominance of self-initiated interventions reflects both role autonomy and the ability to identify and proactively address clinical needs. These findings suggest that expanding NP integration into rehabilitation and other subacute care settings may enhance the quality, efficiency, and continuity of care in Japan’s evolving healthcare landscape. Future research should examine the impact of NP practice on measurable patient and system-level outcomes and explore strategies to optimize NP role implementation across diverse rehabilitation contexts.
